# Reflections on the process, challenges, and lessons learned conducting remote qualitative research on Violence against women during COVID-19 pandemic lockdown in South Africa

**DOI:** 10.1186/s12889-023-17480-z

**Published:** 2024-01-02

**Authors:** Pinky Mahlangu, Mercilene Tanyaradzwa Machisa, Rachel Jewkes, Andrew Gibbs, Nwabisa Shai, Yandisa Sikweyiya

**Affiliations:** 1 South African Medical Research Council, Gender and Health Research Unit, Pretoria, South Africa; 2 School of Public Health, Faculty of Health Sciences, University of the Witwatersrand, Johannesburg, South Africa; 3 School of Health Systems and Public Health, Faculty of Health Sciences, University of Pretoria, Pretoria, South Africa; 4 Department of Psychology, Faculty of Health and Life Sciences, University of Exeter, Exeter, EX2 4QG UK; 5 Institute of Global Health, University College London, London, WC1E 6BT UK; 6 University of KwaZulu-Natal, Centre for Rural Health, Durban, South Africa

**Keywords:** COVID-19 pandemic lockdown, Remote data collection methods, Violence against women research, Intimate partner violence, Telephone interviews, South Africa

## Abstract

**Background:**

Violence against women (VAW) research is a sensitive topic, which has been conducted mainly using face-to-face methods. The COVID-19 pandemic lockdown and restrictions on movement presented an opportunity to conduct VAW research using remote methods. We discuss how we adapted methods, reflect on lessons learned, and make recommendations highlighting key considerations when conducting remote research on a sensitive topic of VAW.

**Methods:**

We designed and conducted an exploratory qualitative study using remote methods with 18 men and 19 women, aged 18 years and older, who lived with their partner or spouse during lockdown in South Africa. The aim of the study was to explore experiences of COVID-19 lockdown, and its link to women and children’s experiences of violence in the homes. Data presented in this paper draws from researchers’ reflections drawn from debriefing sessions during the research process, and from participants’ interview transcripts.

**Findings:**

Remote recruitment of participants took longer than anticipated, and we had to re-advertise the study. We could not ensure safety and privacy during interviews. Regardless of all the safety and privacy measures we put in place during the research process, some participants had an adult person present in the room during interviews, and the researchers had no control over interruptions. Rapport was difficult to establish without an in-person connection, which limited disclosure about violence experience (amongst women) and perpetration (amongst men).

**Conclusions:**

Given the methodological and ethical challenges which limited disclosure of VAW remotely, we conclude that telephone interviews used in our study impacted on the quality of study data. Therefore, we do not recommend VAW research to be conducted remotely, unless it is essential and participants are already known to the interviewer and trust has been established.

## Introduction

Violence against Women (VAW) is a sensitive research topic requiring a skilled interviewer to establish rapport between researcher and participants, as well as ensuring confidentiality, privacy and safety [1]. Due to the importance of these conditions, the field has favoured conducting VAW research using in-person data collection methods to ensure that the research adheres to the required ethical and methodological guidance [1]. However, the COVID-19 pandemic posed challenges for in-person data collection due to the enforced lockdown. On the night of the 23rd of March 2020, the South African government announced a 21-day national lockdown to try and contain the corona virus outbreak as the number of confirmed positive cases jumped from 128 to 402. The 21-day national lockdown, described as alert level 5 started on 26 March to 16 April 2020, further extended until 30 April, thereafter followed by other subsequent alert levels characterized by a gradual easing of restrictions. During Alert level 5, physical distancing measures were implemented including restriction of movement of persons and goods, people could only go outside of their homes to seek or provide essential services and to get food. Most workplaces and schools were closed, leisure and social activities were restricted, and all interprovincial and international travel was banned [2]. The lockdown had negative socio-economic impacts on families and exacerbated some of the known drivers of domestic violence, including loss of livelihood, food insecurity and increased stress [3]. As families spent more time at home than usual, there were emerging reports based on police data and data from call centres in some countries suggesting increased violence against women and children during COVID-19 lockdown [4, 5].

Against this background, primary research was needed to understand the impact of the COVID-19 pandemic on families during lockdown. Qualitative research was important to capture the lived experiences and meanings of COVID-19 pandemic and lockdown for families [6, 7]. Conducting VAW research during COVID-19 was essential to inform violence prevention and response interventions at the time, and for future pandemic preparedness [8, 9]. Asking women to share their experiences during lockdown was crucial in order to gain insights and inform design of responsive policies, services, and programmes [9]. The lockdown and physical distancing measures to control the spread of the virus meant that researchers could not access participants and conduct research in-person. The pandemic and lockdown presented researchers with an opportunity to adapt and use remote methods to conduct qualitative research, while adhering to rigorous ethical guidelines for conducting health research. Adaptation of methods from in-person to remote methods needed to be conducted carefully, especially amongst those working on sensitive topics such as violence against women (VAW), where trust between researchers and participant is crucial to enable disclosure. While we know much about use of conversational face- to-face methods in conducting research on VAW, the use of remote methods to conduct violence research is an emerging area [9]. At the time when research was conducted there were guidelines on how to conduct clinical trials during lockdown [10, 11], but there was limited information on how to conduct qualitative research [12, 13]. The lockdown thus required innovation and adaptation of the design and conduct of qualitative research using remote methods [14].

The literature examining whether, and how VAW research can be safely conducted remotely has started to emerge more recently [8, 9, 15], mainly from research in high income countries [15, 16]. This paper aims to contribute to the evidence drawing from our experience of conducting remote qualitative research with men and women on intimate partner violence during lockdown in South Africa. We were interested to understand whether, and how the lockdown impacted or was linked to women and children’s experiences of violence in the homes in South Africa. The findings of the study are published elsewhere [3]. In this paper, we discuss how we adapted methods in order conduct VAW research remotely during COVID-19 lockdown, and reflect on ethical and methodological challenges we experienced while conducting VAW research using remote methods to recruit participants, collect data, and disburse reimbursements for time. We further make recommendations and highlight key considerations for conducting remote research on sensitive topics. The framework we use to structure the findings is the research process: from recruitment of participants, getting informed consent, and data collection. The paper contributes to the growing literature on use of remote methods on sensitive research topics in public health.

## Methods

### Study design and site

The study was conducted amongst 18 men and 19 women, aged 18 years and older, who lived with their partner or spouse during lockdown, in Gauteng province - one of the nine provinces in South Africa, which had the highest number of COVID-19 cases during the pandemic. Gauteng is the smallest of the South Africa’s nine provinces, but an urban and economic hub of the country which consists of cities including Johannesburg, Tshwane, Ekurhuleni and other surrounding metropolitan areas [17].

### Recruitment

We developed and posted the study advert inviting anyone who was eligible to participate on the South African Medical Research Council’s (SAMRC’s) Facebook page, and shared it on personal Facebook accounts of the study team. The advert was also shared with social networks via WhatsApp by the research team. We encouraged social networks to widely share the advert with their networks. The study advert invited participants to share their experiences of the COVID-19 pandemic and lockdown. It had an email address and a cellphone number, which prospective participants used to contact the researchers to indicate interest to participate in the study. After receiving a message from participants indicating interest to participate, the study team would make initial contact with prospective participants using voice call. The initial call was to explain the objectives of the study, data collection procedures, potential risks, and benefits of participation, and to screen participants for eligibility. Furthermore, the importance of privacy and safety when conducting the interview was explained to participants, those who indicated that they had a private space and time to conduct the interview were included in the study.

To be eligible to participate in the study one had to be a man or woman, 18 years and older, living in Gauteng for an uninterrupted period between February and July 2020 during lockdown, living with a spouse and/or child(ren). In addition, we asked participants to indicate their income bracket, and used Statistics South Africa annual household income classification to categorize participants into low income, or middle and high income groups [18].

### Ethical considerations

Ethics approval to conduct the study was granted by the SAMRC Human Research Ethics Committee (EC008-5/2020), and the research adhered to the WHO’s ethical standards for conducting VAW research [1]. Amongst others, there were four key ethical considerations we complied with when conducting the research remotely: we emphasized obtaining informed consent, the importance of privacy and safety during interviews, ensuring that the telephone interviews were conducted by experienced researchers with skills on conducting VAW research, and having measures to refer those who reported experiences of gender-based violence (GBV) to services. Whilst the WHO Guidelines on Ethical and Safety Recommendations for Research on Domestic Violence Against Women (2001) were useful to inform how we designed and conducted the research, they were limited in that they were developed to guide VAW research using face-to-face survey methods. Thus, we needed to adapt some of the methods to be suitable for conducting VAW research remotely. The Table 1 describes these adaptations.

**Table 1:** Procedures and adaptations of methods for remote recruitment and data collection

WHO defined safety and ethical recommendations areas for research on VAW	Traditional/In person	Remote adaptation
Recruitment and getting informed consent	Recruiters would approach participants in person, inform them about the study, invite them to participate, get written consent, and conduct the interview.	Participants were recruited remotely using social media platforms. The study advert was posted on Twitter, Facebook and shared with networks on WhatsApp. Potential participants were asked to contact the cellphone number provided on the advert to express interest in participation. The PI called interested persons, explained the study and screened for eligibility using tool developed for study, then sent eligible participants study information sheet and consent form to fill and return back using WhatsApp. When consent form was returned, interview appointment was set.
Interview scheduling, privacy and safety during interview	The need and importance of privacy and safety is explained by the researcher. The time and suitable location that allows for privacy and safety (no third person can overhear, unless it’s a child under 12, and there is limited or no interruptions) during the interview is selected and agreed by both the researcher and participant.	The need and importance of privacy and safety is explained by the researcher. Interview time guided by the participant’s availability for a private interview over the phone is selected. Space to sit and do the interview is determined by participant based on their personal home circumstances. The researcher has no control over interruptions during the interview. Both agree before the interview on measures to take when privacy and safety is compromised. In our study, we agreed that participants will hint to the researcher by talking about the weather whenever privacy and safety has been compromised.
Rapport, detecting and dealing with participant distress	In person engagement which involves eye contact and handshake helps facilitate the process of relationship building and establishing rapport between researchers and participants. However, adequate training of and interviewing skills of the interviewer are also important in enabling the interviewer to build connection and establish rapport.	The phone limits the ability to establish a connection without in person contact. The researcher relies only on voice and tone (interrupted by network and poor connectivity), with no visual cues. To establish rapport, we started the interview by having an informal conversation with participants, sharing information about ourselves and families, and started the interview by asking participants about their family, who they stay with, how many children they have, how old the children are, and whether the children go to school. From the start of the interview, we were attentive, showed interest in what the participant was sharing, and asked questions, highlighted where there were mutual interests and similarities about family, as a way to establish a relationship with participants.
Reimbursements	Information about reimbursement is included in the information sheet. The researcher is able to give the reimbursement directly to participants, who can confirm receipt by their signature.	The reimbursement was not disclosed in the information sheet, only after the interview. Only money for data, to enable WhatsApp communication was disclosed. This was done in order to limit the chances of knowing about reimbursement influencing participant’s decision about participation. The reimbursement was sent remotely on e-wallet, and the researcher had limited control or way of verifying that participants directly received the reimbursement.

Participants were given an opportunity to ask any questions and thereafter asked to provide verbal and written informed consent using WhatsApp, phone text or email. A consent form was sent to participants on the platform they selected for communication, most opted for WhatsApp, but a few preferred an email. The consent form was in English, had two brief questions, which had responses “yes and no”, asking participants “Do you agree to participate in the study?” and “Do you agree for the telephone interview to be audio recorded, and data to be anonymously used for the purpose of the study?”. Written consent received from participants was transferred to an email, then downloaded and safely stored on a password protected computer. The initial call would end with setting-up an appointment for the interview on the day and time suitable to participants, when they will have privacy to take the call.

### Remote data collection process

Data was collected by some members of the research team, who are co-authors of the paper. The research team has extensive experience in conducting in-depth interviews (IDIs) on gender-based violence in South Africa and other settings. We conducted in-depth telephone interviews (IDTIs) of between 35 and 60 min, with 37 participants (18 men and19 women). The telephone interviews were conducted in order to comply with the COVID-19 social distancing measures to protect both the researchers and the participants. The interviews were conducted using a semi-structured interview guide with open-ended questions, and the interviewers were matched by gender and in the language preferred by the participants. Most of the interviews were conducted in English, and a few in IsiZulu, Sepedi, and IsiXhosa, the three predominant vernacular languages in Gauteng province. We used a digital PR200 cellphone recorder, which allowed for both calling and recording of the interview on one device. Reimbursement of R100 ($7.13) was given to all participants after the interview for their time, and R30 ($1.66) for data.

### Debriefing sessions

The reflections presented in this paper are drawn from debriefing sessions that the research team had during data collection and at data analysis stages. The paper also draws data from the transcripts of interviews with participants to support or illustrate some of the reflections presented. The research team met three times during data collection process to reflect on the data collection process, discuss what was coming-up from the interviews, and to provide one another with emotional support during the lockdown period. Two meetings were also held by three members of the research team who coded the transcripts, developed the codebook and defined emerging themes, which were after shared with the whole research team for deliberation. The purpose of the two meetings was to share and discuss the codes and emerging themes from the data which were individually coded by the three co-authors. During the discussion, the coders further shared their reflections, and highlighting areas in the transcripts which were addressed in detail by participants, and those that were not. Reflections were also shared by the team during the writing of the study findings, where all co-authors provided written comments on the manuscript of the paper published, which were incorporated by the corresponding author [3]. The study PI took notes from the debriefing sessions and coding meetings held, which included reflections of all the co-authors except RJ who was only involved in the writing of the paper. Cross-team member discussion of the final set of reflections was done during conceptualization and writing of the paper.

## Results

The research process is used to structure the findings, starting with recruitment of participants and getting informed consent, and data collection using remote telephone interviews.

### Recruitment of participants and getting informed consent

We remotely recruited participants from a wide age range, including older women and men. Participants were aged between 24 and 62. However, we observed and discussed that remote recruitment of eligible participants through online platforms took longer than we have experienced when conducting in-person recruitment. Moreover, we had to re-advertise the study before receiving sufficient expressions of interest from people who met the criteria for participation in the study. The long-time taken to recruit and the need to re-advertise the study suggest that either remote recruitment in our study was not a time-efficient method of recruitment or the criteria for the study and the platforms used may not have been readily accessible or as popular as we anticipated to the target population. We reflected that while social media allowed for a wider reach, our recruitment strategy might have excluded people who do not use social media platforms. It is also possible that some people who met the study criteria may have been hesitant, skeptical, or suspicious about participating in the study and this deterred their expression of interest. When participants are recruited in person, there is an opportunity for them to voice their concerns about the study and seek clarity by asking questions to recruiters.

### Data collection using remote telephone interviews

Telephonic interviews were conducted at a second telephonic engagement with the participants, after receiving written informed consent. A few participants (about 8%) rescheduled their interview when we called them. Most of the interviews were conducted on the date and time selected by participants. This suggested that most participants found it easy to fit the interview into their schedule. We had a few instances where we had a bad line or network connectivity challenges, and the call kept cutting. Three key areas of learning were noted during the telephone interviews: establishing rapport, privacy during interviews, and disclosing violence experience and perpetration over the phone.

### Rapport during telephone interviews

As researchers who conducted remote telephone interviews we learnt that rapport was difficult for us to establish without an in-person connection. While the first few questions in the interview guide were designed to build rapport (e.g. asking participant’s background information and about their families), it was difficult for us to establish rapport with participants over the phone. However, this experience varied. Some participants were open and willing to share broadly about their lives, while others were reserved and shared very little during the interview. To obtain information from those less open, we probed and asked the questions differently, however, this also did not yield much information. Another limitation with telephone interviews was not being able to see visual cues, which limited probing. We were sensitive to not probe to a point of causing distress, recognizing that we did not have visual cues to enable us to detect distress.

### Privacy during telephone interviews

Before we commenced with the interview, participants were asked if they were in a private space and safe to conduct the interview. We proceeded with the interview only when participants confirmed that they were in a private space, and that it was safe to conduct the interview. Strategies used to ensure privacy varied, with some of the participants, for example, conducting the interview in their car. Further, participants were asked to indicate to the interviewer when their privacy was compromised during the interview, given that the researcher was not physically in the room with them to be able to monitor this. The interview guides were designed to ask open ended questions, for example, how participants were affected by COVID-19 pandemic, and how they experienced the COVID-19 lockdown in their families. We framed the questions in this manner to allow participants privacy to share what they wanted to share, although there was follow-up probing from the researchers. Throughout the interview, the interviewer ensured that they listened for signs of discomfort in participants’ responses, and tried to determine from the tone when there was distress and not probe further. For example, when a participant said “I would rather not talk about that in detail”. We assumed in those instances that the participant did not feel safe to discuss the matter further and did not push the line of inquiry. After such an instance, we would ask if the participant was still okay to continue or move to the next question.

During some interviews we realized that some participants did not have the privacy, that they had indicated prior to the start of the interview. For instance, we could hear voices in the background which suggested that a third adult person was present in the room during the interview. In some interviews with women, we heard a sound of a baby crying, with some women requesting to be excused to give the child to another adult person who was present at home. We could not ascertain whether that adult person was in the same room with the participant or a different room. One male participant could not openly respond to a question about whether there was any violence between him and his spouse in the home during lockdown. Limited by the presence of his spouse in the room, he responded:

It once happened to me, but my wife is here right now.

We learnt in our study that as researchers we have limited ability to ensure privacy during remote telephone interviews with participants. This is an area requiring further exploration to find ways to improve to ensure safety of participants.

### Reimbursement of participants after telephone interviews

Shifting to remote methods also required that we adapt how we reimbursed participants for time. We needed to find an accessible platform to send money to participants without physical contact. We sent reimbursements using a digital e-wallet, which required participants to have a mobile phone to receive a pin number from the bank to use to withdraw money from an ATM, whenever is convenient to them. The pin number is easily renewable on one’s mobile phone at no cost to participants. With widespread ownership of mobile phones in South Africa, use of digital e-wallet to send and receive money is common across all socio-economic groups. E-wallet is considered a convenient method of receiving money as it does not require one to have a bank account, and the bank charges are charged to the person sending the money. The reimbursements were only disclosed to participants at the end of the interview to ensure that they do not influence decision about participation in the study. We did not know how easy or difficult it was for participants to access the money, but none reported having experienced challenges.

### Disclosing violence experience and perpetration during telephone interviews

Our analysis of data revealed that talking about experiences and perpetration of intimate partner violence over the phone was challenging for most participants, both male and female, in varied ways. Both male and female participants spoke openly and elaborated in their responses about how COVID-19 and lockdown affected their livelihoods, difficulties of parenting during lockdown, violence they perpetrated against children, and about high levels of stress they experienced. However, both male and female participants were hesitant to respond to intimate partner violence (IPV) questions. Most women avoided talking about personal experiences, and more openly talked about IPV experiences of others in their family or community:

I wouldn’t say its violence; my husband is short-tempered and has anger. My next door neighbour… there was violence between him and the partner there. He is staying with a girlfriend. They are always fighting to an extent that those people break everything in the house.

“My parents have had episodes of violence during the lockdown I would say. My dad has definitely uhm I think he’s starting to feel very cooped up and cranky, very impatient with my mom, aggressive and I wonder what else happens. We [husband and I] only had disagreements, sometimes very emotionally draining. My husband is a good husband.”

Amongst women who reported experiences of violence in the home during lockdown, only a few reported experiences of physical violence. Most women reported emotional violence perpetrated by their spouses.

Some men understated their perpetration of IPV during lockdown, avoiding labelling their experiences as violence, rather spoke about “small disagreements”, “misunderstanding”, or “tension”:

I am working in a food store, my wife doesn’t work, sometimes when i get home you find there is no food, sometimes she would blame me and pressurize me, you understand, we end up having a misunderstanding… she was saying you are the father, you should make a plan, you understand, she was giving me pressure. Sometimes we used to fight physically and breaking things in the house and sometimes verbally, but it was nothing much.

Being confined in one space with someone that you are not used to spend much time together - because you were used to wake up in the morning and leave the house for work - that caused so much conflicts. We used to have a lot of small fights.

There was tension, mainly caused by her moods, we would have heated exchanges, but I would not touch her.

A number of participants, both men and women, openly reported having beaten, shouted or threatened their children during lockdown. Participants in our study found it easier to talk about violence against children (VAC) than intimate partner violence. This could be explained by dominant beliefs about the acceptability of corporal punishment of children at home in South Africa.

## Discussion

This paper discusses adaptation we made, reflections, and lessons learned on using remote methods to conduct VAW research during COVID-19 lockdown in South Africa. Our study has confirmed some of the critiques of remote data collection documented in the literature suggesting underreporting, and difficulty of establishing rapport and ensuring participant safety and privacy without in-person interaction [8, 15]. Our data also highlight some positive findings around the ease of scheduling telephone interview appointments, which participants found easy to fit into their schedule. Most of the scheduled interviews were conducted on the date and time agreed upon during the first call, and very few participants rescheduled their appointments. We could not secure privacy during the interviews, and this impacted on data quality. Even with the measures we implemented, before and at the start of the interview, we had no control over participants’ environments. As such, the lack of privacy limited participant’s ability to talk about experiences and perpetration of violence, which raised questions about validity and credibility of the data. There is acknowledgement in the literature that research methods that do not involve face to face engagement with participants and restrict the researcher’s ability to access their natural environment may limit the depth and extent to which researchers can explore the topic under investigation [12].

The safety of women participating in research on violence is extremely important and emphasized in the WHO Guidelines on Ethical and Safety Recommendations for Research on Domestic Violence Against Women [1, 9]. While safety and privacy of participants was amongst the key ethical considerations in our research, it was a challenge to prevent disturbances during remote interviews with participants. Even after we had discussed the importance of privacy and stopping interviews if interrupted during interview, participants did not inform us as someone came into the room, and we had no other way of learning about this as we were not there. We had to rely on participants to manage their own safety risks around disclosure and the conversation and that was not satisfactory as we don’t know how well they did it. Our study further confirmed what has been observed in other studies which showed that talking about violence experience and perpetration is highly sensitive and difficult over the phone [8, 9]. Without in-person contact, rapport between the interviewer and participant, which is critical for enabling disclosure of personal information, was difficult to establish. Participants in our study were reluctant to engage on personal topics and disclose violence experience (women) and perpetration (men). It was evident in some of the extracts that a few participants could not speak openly, and feared being overheard by their spouses, whom in some instances were present in the room during the telephone interview. As such, only a few women disclosed and reported experience of physical violence in our study. Many were reluctant to talk about personal experiences of violence. It could be that there was more violence experienced and perpetrated during lockdown than disclosed in our study. The two telephone calls or remote interaction with participants might have not been adequate to establish rapport and trust necessary to enable participants to openly and safely share about sensitive personal experiences. More research is needed to deepen understanding on how to remotely establish rapport and create participants’ emotional safety, and physical privacy to enable openness to share about sensitive topics during telephone interviews.

Notwithstanding, we also acknowledge that there could have been other factors such as social desirability and cultural beliefs that might have limited disclosure of violence experience and perpetration during COVID-19 lockdown amongst men and women in our study. At the time of the study, there was media coverage, and political calls and messaging on violence against women and children in South Africa. GBV was described by the President of the country as a second epidemic facing the country, with widely publicized messaging against it [19]. This meant that sensitization against GBV was high and men in our study may have not wanted to be counted amongst those who were perpetrating, and women as survivors of GBV. Furthermore, there are beliefs or socialization in some cultures in South Africa where disclosing what happens in a marriage or relationship with a spouse is discouraged. Everything about the marriage or relationship including IPV is considered a domestic matter, that needs to be kept private. Women who reported experiences of violence were referred by us to contact the National GBV Command Call Centre and the police. However, we do not know whether such referrals were taken up by the women.

Social media was possible to use to get participants, but it was not a very satisfactory way of recruiting people as we used multiple platforms and had to advertise twice. This suggests that the response rate was low and it was very hard to know what sort of biases would have been introduced. However, the value of using social media platforms for recruitment of participants in health research is increasingly being recognized for their wide reach, given their accessibility and use by most people [20–22]. Others have found social media to be an effective and cost effective platform which allowed for reach of a large number of participants during COVID-19 [23]. Despite the merits of social media platforms, others have cautioned that social media platforms can be biased towards a younger sample of participants from high socio-economic status, which was not the case in our study [20, 24]. Our sample included both young and old men and women between age 24–62 years, from low, middle and high-income groups, with some unemployed. We had slightly more [19] females compared to [18] male participants in our study. We were very concerned about not overly inducing participation by saying that participants would be reimbursed for participation. As a result, unlike in other research, we did not tell participants this in advance. This may have contributed to the slow rate of recruitment and possibly some people who would have liked to earn something and share about their experiences didn’t do so as they didn’t know they would get reimbursement. Getting written informed consent on WhatsApp or SMS from participant’s in remote research was possible, though relatively new in health research, it is increasingly being accepted by Human Research Ethics Committees [25]. Others have argued that remote consent from participants who self-select to participate is truer and non-coercive given that participant’s express interest without researchers’ putting undue pressure on them to participate [26].

To respond to the challenges of establishing rapport, research on sensitive topics like violence can be conducted remotely only when participants are already engaged in the study and the partnership between participants and researcher is well established [8, 25, 27]. An established relationship of trust between participants and interviewer is important and can be used as leverage to facilitate rapport and connection in a remote interview. Starting with an informal conversation and asking questions that are not personal to the interviewee is also crucial to build the connection [25]. Bhatia et al. (2022) further suggest that shifting to remote data collection in violence research requires additional safeguarding processes, remote support for researchers, committing resources to additional steps required to protect participants adapting to moveable and unpredictable conditions specific to each context.

One needs to have a well-designed study, with clear safety and privacy protocols for participants. While the researcher has limited control of the space, he/she should before and at the start of the interview explain safety and privacy requirements to participants, and why that is important. There is a need to guide interviewers on how to respond or make safety decisions in the event where privacy is breached and women’s safety is compromised during a telephone interview. Use of safe words or code words by participants to indicate when safety is breached is encouraged for remote research on violence [27]. Emotional safety is important for women to share their experiences on sensitive topics. Given that there are no verbal cues to assist the interviewer to detect distress from participants telephone interviews, it is important that the interviewer carefully listens to detect changes in the tone and sudden pauses which can be interpreted to signal distress. Should the interviewer detect distress, they should always remind participants that they do not have to respond if not comfortable, and of their right to withdraw at any point during the interview. Remote interviews should be conducted either by experienced researchers, or well-trained interviewers who will know when and how to probe, implement privacy and safety protocols, and be able to detect distress over the phone. Debriefing sessions to support the research team during data collection, and to provide them space to brainstorm solutions to challenges encountered is even more critical when conducting remote research. Safeguarding and referral of participants to support services remains crucial in all research on sensitive topics, online or phone based services are most useful when movement of participants is restricted [25]. Our research had some limitations in that the study was conducted amongst a sample of men and women in Gauteng and thus the findings are not generalizable. However, the lessons learned in this study may be useful for others conducting remote research on sensitive topics in similar contexts. We acknowledge the limitations of the remote recruitment strategy we used, which might have excluded people who do not use social media platforms including Facebook and WhatsApp.

## Conclusions

The COVID-19 lockdown and restriction of movement presented an opportunity to explore and use remote methods to conduct qualitative research on a sensitive topic of violence against women. In our study, we learnt that conducting VAW research using telephone interviews is challenging presents with methodological and ethical challenges around privacy and safety. It was difficult to establish rapport with participants without in-person contact, and negatively impacted on disclosure of violence experience and perpetration. Whilst these challenges are present in face to face research, they were heightened in remote research. Whilst the WHO guidelines on conducting domestic violence research with women, which we used in designing our study, were useful, they were limited at the time, as they were not designed for remote research. We conclude that conducting qualitative VAW research remotely requires a well thought through study design and planning; should be done by skilled researchers or well-trained interviewees, guided by privacy and safety protocols; among participants where a relationship of trust has been established, and have a clear understanding of the importance of safety and privacy during an interview. Lessons learned in designing and implementing remote methods during COVID-19 lockdown in our study could be useful for others planning to use similar methods to research sensitive topics, and is a contribution to knowledge on execution of remote research in public health.

## Data Availability

The datasets analysed in the study are available from the corresponding author on reasonable request.

## List of abbreviations

ATM: Automated Teller Machine
CoE: Centre of Excellence
GBV: Gender Based Violence
IDTI: In–depth Telephone Interviews
IPV: Intimate Partner Violence
NRF: National Research Foundation
PI: Principal Investigator
SAMRC: South African Medical Research Council
WHO: World Health Organization
